# Natalizumab Treatment for Relapsing Multiple Sclerosis Stabilises Normal-Appearing White Matter Microstructure: A One-Year Prospective Ultra-High-Field Quantitative Imaging Study

**DOI:** 10.3390/brainsci13101464

**Published:** 2023-10-17

**Authors:** Radu Tanasescu, Olivier Mougin, I-Jun Chou, Ali Al-Radaideh, Oltita P. Jerca, Su-Yin Lim, Penny Gowland, Cris S. Constantinescu

**Affiliations:** 1 Academic Unit of Mental Health and Clinical Neurosciences, Section of Clinical Neurology, University of Nottingham, Nottingham NG7 2UH, UK; 2 Department of Neurology, Nottingham Centre for MS and Neuroinflammation, Nottingham University Hospitals NHS Trust, Nottingham NG5 1PB, UK; 3 Sir Peter Mansfield Imaging Centre, School of Physics & Astronomy, University of Nottingham, Nottingham NG7 2QL, UK; olivier.mougin@nottingham.ac.uk (O.M.);; 4 Chang Gung Memorial Hospital, Linko Branch, Taoyuan 333, Taiwan; 5 Department of Medical Imaging, Faculty of Applied Medical Sciences, The Hashemite University, Zarqa 13133, Jordan; 6 Department of Medical Radiography, College of Health Sciences, University of Doha for Science and Technology, Doha 24449, Qatar; 7 Medizinisches Zentrum Harz, 38820 Halberstadt, Germany; 8 School of Medicine, Faculty of Health and Medical Sciences, Taylor’s University, Subang Jaya 47500, Malaysia; 9 Cooper Neurological Institute, Cooper Medical School of Rowan University, Camden, NJ 08013, USA

**Keywords:** multiple sclerosis, ultra-high-field MRI, natalizumab, normal-appearing white matter

## Abstract

(1) Background: Natalizumab dramatically reduces relapses and MRI inflammatory activity (new lesions and enhancing lesions) in multiple sclerosis (MS). Chemical exchange saturation transfer (CEST) MRI can explore brain tissue in vivo with high resolution and sensitivity. We investigated if natalizumab can prevent microstructural tissue damage progression measured with MRI at ultra-high field (7 Tesla) over the first year of treatment. (2) Methods: In this one-year prospective longitudinal study, patients with active relapsing–remitting MS were assessed clinically and scanned at ultra-high-field MRI at the time of their first natalizumab infusion, at 6 and 12 months, with quantitative imaging aimed to detect microstructural changes in the normal-appearing white matter (NAWM), including sequences sensitive to magnetisation transfer (MT) effects from amide proton transfer (MTRAPT) and the nuclear Overhauser effect (MTRNOE). (3) Results: 12 patients were recruited, and 10 patients completed the study. The difference in the T1 relaxation times at month 6 and month 12 of natalizumab treatment was not significant, suggesting the lack of accumulation of tissue damage, while improvements were seen in MTR (MTRAPT and MTRNOE measures) at month 12, suggesting a tissue repair effect. This paralleled the expected lack of clinical and radiological worsening of conventional MRI measures of disease activity (new lesions or gadolinium-enhancing lesions). (4) Conclusion: Natalizumab prevents microstructural brain damage and has effects suggesting an improved white matter microstructure measured at ultra-high field during the first year of treatment.

## 1. Introduction

The humanised monoclonal antibody natalizumab (Tysabri^®^) is a highly effective drug for relapsing–remitting multiple sclerosis (MS). Natalizumab dramatically reduces clinical relapses and MRI-detected MS active lesions (new T2 lesions and gadolinium-enhancing lesions). Natalizumab also prevents, to a great extent, the subtle damage of the normal-appearing white matter (NAWM) captured with more advanced imaging techniques such as diffusion tensor imaging (DTI) or magnetisation transfer ratio (MTR) as shown by 1.5 and 3 Tesla MRI studies [1,2]. This may represent reduced blood–brain barrier permeability [3] and/or microglia activation [4]. However, it is unclear if natalizumab effects on stalling NAWM damage in MS can still be demonstrated at ultra-high-field MRI. Compared to low-field MRI, images acquired at ultra-high-field MRI generally have higher signal-to-noise and contrast-to-noise ratios, smaller voxels, and stronger susceptibility contrast [5,6]. Seven-Tesla MRI can provide increased sensitivity to subtle changes in NAWM compared to lower-field MRI [6,7,8], and, therefore, despite technical and logistical challenges, can offer the possibility of improved correlations with neurological disability in MS. The increased sensitivity of ultra-high-field MRI allows images to be acquired with smaller voxels, providing better information about the heterogeneity of any changes occurring in WM. Additionally, the increase in spectral resolution with a higher magnetic field improve the sensitivity of molecular imaging techniques such as amide proton transfer (APT), a particular type of chemical exchange saturation transfer (CEST) MRI that is sensitive mainly on the intracellular mobile peptide and protein concentration and pH, and has been shown to increase the APT signal in MS lesions compared to normal WM [9]. Similarly, another magnetisation transfer (MT) method called a relayed nuclear Overhauser effect (rNOE) can help in identifying multiple sclerosis lesions due to its sensitivity to protons in lipids mainly associated with myelin in the brain [10]. Both methods are additional to the MT phenomenon measured via MTR, but only visible at specific frequencies. In MS, an increase in APT may be caused by changes in the intracellular amide proton content [9] and the increased protein degradation during axonal damage with secondary higher concentrations of mobile peptides [11,12]. Similarly, a decrease in relayed NOE contrast has been reported in MS lesions and to a lesser extent in the white matter compared to healthy controls, indicating the sensitivity of rNOE towards myelin change [10].

T1 relaxation time is a quantitative MRI measure that is sensitive to water binding and tissue microstructure, and MTR is proposed as measure of myelination [13]. We have previously shown that ultra-high-field MTR measures are sensitive to changes in NAWM in early MS [14] and are a robust alternative for detecting demyelinating lesions, comparable to 3 T FLAIR [15]. The pattern of NAWM anomalies detected with MTR at conventional MR field strengths may precede the development of T2 lesions by up to two years [16]. Several disease-modifying treatments in MS can avert the longitudinal decline in MTR measures and may potentially prevent the accumulation of microscopic damage in the brains of MS patients [17,18].

We aimed to use quantitative ultra-high-field MRI to assess if natalizumab is effective in preventing any NAWM change in active MS, in the first year of treatment. We longitudinally assessed the NAWM using T1 relaxation time and two different MT measures: one sensitive to standard macromolecule MT effects as well as amide proton transfer (MTRAPT), and a second sensitive to macromolecule MT effects as well as the relayed nuclear Overhauser effect (MTRNOE).

## 2. Materials and Methods

### 2.1. Patients and Study Design

Twelve patients with the diagnosis of MS according to the McDonald criteria revised 2010 [19] with active relapsing-remitting MS were recruited between 2012 and 2015 from the MS Clinic at the University Hospitals Nottingham. All patients were due to start treatment with natalizumab intravenously monthly, as per their routine MS care, according to NICE (National Institute of Health and Clinical Excellence, UK) 2010 guidelines. The patients had to have rapidly evolving active MS, with two disabling clinical relapses in the prior year. Patients had not received systemic glucocorticoid or other disease-modifying treatment in the 3 months preceding the initiation of the treatment and had not experienced any clinical relapses during these three months. Additional exclusion criteria were planned pregnancy, systemic comorbid disease, and exposure to immunosuppressive treatment in the three months before the study.

This was an exploratory study. The sample size was based on our prior work [14] showing that the distinction of NAWM changes between controls and different severity MS groups with this imaging protocol was possible with 10 patients per group. We recruited 12 subjects to anticipate and cater for the risk of dropout or missing data. This sample size was used in our exploratory studies of the immunological effects of natalizumab on T regulatory cells [20].

The study was approved by the East Midlands Research Committee, England (11/EM/0341). All patients gave written informed consent.

Patients were assessed clinically and radiologically on the day for their intravenous treatment with natalizumab (before the infusion), at baseline (visit 1, V1—the day of first natalizumab infusion), month 6 (V2), and month 12 (V3). At each visit, interval history was obtained, with particular attention to side effects, missed treatments, and relapses. The patients underwent a neurological examination with an estimation of their expanded disability status scale (EDSS) scores (Kurtzke 1983).

### 2.2. MRI Data Acquisition

MRI scanning was performed on a 7 T Philips Achieva system, equipped with a 32-channel receive coil and head-only volume transmit coil at Sir Peter Mansfield Imaging Centre.

The following images data sets were acquired:

(1) To produce T1 maps, seven 3D inversion recovery TFE (IR-TFE, or MPRAGE) images were acquired across the deep grey matter at a range of inversion times (150, 300, 500, 800, 1200, 1800, 2500 ms). The acquisition parameters were as follows: TE = 3.2 ms; TR = 6.9 ms; flip angle of the TFE readout pulse = 8°; TFE factor per inversion = 240; shot-to-shot interval = 8 s; spatial resolution = 1.25 × 1.25 × 1.25 mm^3^; field of view = 200 × 200 × 72.5 mm^3^; scan time per TI = 2 min. An adiabatic, phase-modulated pulse with a bandwidth of 1.6 kHz and duration of 13 ms was used for inversion [21].
(2) MT-weighted images were acquired using a 3D MT-prepared turbo field echo (MT-TFE) sequence (0.86 × 0.86 × 1.5 mm voxel size). Three acquisitions were made: one with no presaturation, one with presaturation −1.05 kHz off-resonance from water (MTRNOE: sensitive to MT and NOEs), and one with presaturation +1.05 kHz off-resonance (MTRAPT: sensitivity to MT and to a lesser extent to amide proton transfer) (total scan time = 8 min 22 s for the three acquisitions, further details can be found in a prior publication [14]).
(3) High-resolution (0.6 × 0.6 × 0.6 mm^3^) MPRAGE (T1-weighted) anatomical images were acquired prior to and post injection of gadolinium (0.1 millilitre Gadovist per kg body weight). Details of the acquisition were as follows: TE = 5.9 ms, TR = 15 ms, shot-to-shot intervals 3000 ms, turbo field echo (TFE) factor = 148, inversion time = 1050 ms, flip angle = 8°, spatial resolution 0.6 mm isotropic, matrix size 320, pseudo-radial k-space sampling for a total acquisition time of 11 min 40 s.

### 2.3. Data Analysis and Post-Processing

T1 maps were calculated from the IR-TFE images as described previously [22]. The MT images acquired at positive and negative frequency offset were co-registered to the reference scan acquired with no saturation pulse using rigid-body registration with six degrees of freedom in FSL (FMRIB, Oxford, UK). MTR maps were then calculated on a voxel-by-voxel basis for MTRNOE and MTRAPT.

The IR-TFE image that was acquired near the null point of grey matter (GM) was used to segment the GM and WM in SPM (http://www.fil.ion.ucl.ac.uk/spm/). This process defined MS lesions in WM as GM tissue, providing a route to automatic segmentation of WM lesions. The segmented WM was displayed as a probability map, which was converted to a mask and then eroded by two voxels to exclude any partial volume effects at the GM or lesion borders. Hereafter, this mask will be referred to as an NAWM mask.

The reference volume in the MT sequence was then co-registered to the IR-TFE image used to create the NAWM mask, with rigid-body image registration using the FLIRT linear registration algorithm from the FSL platform (FMRIB). Because of the difference in contrast between the IR-TFE image and reference scan, the cost function was the “mutual information” with a low number (100) of bins. The registration matrix was applied to the registered MTRNOE and MTRAPT maps to transfer them into the same space as the IR-TFE image. Finally, the NAWM mask was then used to extract the NAWM tissue values from the T1, MTRNOE, and MTRAPT maps. Both MTRNOE and MTRAPT were found to depend strongly on B1 amplitude, such that the peripheral WM tended to have lower MTR values than the more central parts regardless of any abnormalities. Therefore, the relative effect of B1 field inhomogeneity on MTR values within the NAWM mask was corrected using a method described by Ropele et al. [23].

Histograms of the pixel values within the NAWM mask of these maps were created for each subject, and the median, full width at half maximum (FWHM), peak position (mode), and cut-off for the upper quartile of the T1 histogram and lower quartile of the MTR histograms of the histograms were calculated. A subject-averaged histogram was also produced for each MRI measurement.

Lesion evolution between the three visits was evaluated from the high-resolution MPRAGE images. For each subject at each visit, segmentation into GM, WM, and CSF, as well as abnormal tissue (lesions), was performed using the CAT12 toolbox (http://dbm.neuro.uni-jena.de/cat12). Specifically, for each participant, the scans from all three visits are registered and bias-corrected using the serial longitudinal registration in SPM12. Net tissue change was quantified by measuring tissue volume for each class, normalised to the total brain volume.

### 2.4. Statistical Analysis

All statistical analyses were performed in SPSS 27.0 (SPSS, Chicago, IL, USA). Normality was tested using the Kolmogorov–Smirnov test. One-way ANOVA with repeated measures was performed on parametric data, testing the assumption of sphericity with the Maulchy’s test of sphericity. For pairwise comparison after one-way ANOVA analysis, the tests were adjusted for multiple comparison using Bonferroni correction. Masking and histogram analysis were applied to each repeated data set as above.

## 3. Results

### 3.1. Participants and Clinical Course of MS

A total of 12 participants were recruited in the study (7 females and 5 males), with a mean age of 34.2 years (range 21–45 years, standard deviation SD ± 7.25). The median disease duration was 3 years (range 3–7, SD ± 4.6), and diagnosis of MS was made in the first year from symptom onset. The mean EDSS in this group at baseline was 3.5 (range 1–7, SD 1.8) (Table 1). All patients were oligoclonal-band-positive in the cerebrospinal fluid at diagnosis. All patients had vaccination status as per NHS England protocols, were hepatitis B- and C-negative, were HIV-negative, and did not have any history of cancer nor any associated psychiatric and autoimmune conditions. All patients were vitamin-D-insufficient at diagnosis, with subsequent oral supplementation implemented as per guidelines.

Over the course of the one year of observation, none of the patients experienced a clinical relapse during the treatment with natalizumab. The EDSS score improved in three patients (3 to 1.5; 5 to 4; and 7 to 2) between V1 and V3. None of the patients in this study experienced allergic reactions/infusion reactions to natalizumab, or opportunistic infections during the study.

After the end of the study, the patients were continually followed-up in clinic and with 6 m or yearly MRI scans (1.5 T or 3 T) up to 10 years from study baseline (last patient follow-up: July 2023). Five out of twelve patients carried on treatment with natalizumab at ten years from study onset with very good disease control. In four, EDSS was stable at 10 years, and, in one patient, EDSS was stable at five years and increased by 0.5 points at 10 years, presumably due to PIRA (Progression Independent of Relapse Activity). In the remaining seven patients, treatment with natalizumab was switched after at least three years of treatment to another approved MS therapy: in four patients as a de-risking strategy (seroconversion to JCV-antibody-positive status and subsequent increase in the risk of PML) and in three for other reasons (pregnancy, relocation, patient preference). None of the 12 patients experienced clinical relapses during the natalizumab treatment during this follow-up. There were no cases of PML in this group.

### 3.2. MRI Analysis

**Figure 1:**
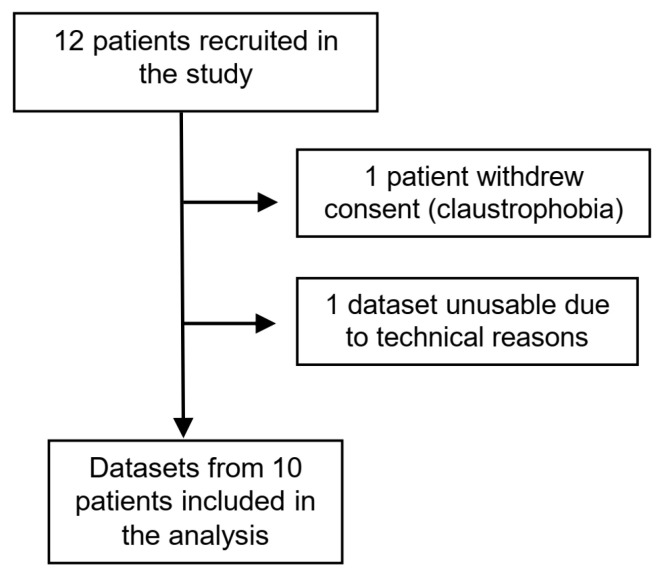
Flowchart of participants in the study.

Analysis was performed on 10 datasets (nine complete V1–V3 datasets and one V1–V2). Data for one patient were unusable for technical reasons. One patient attended V1 and V2, then stopped natalizumab due to pregnancy; datasets were included in the analysis. One patient withdrew consent and pulled out of the study after the first visit (claustrophobia) (Figure 1).

**Figure 2:**
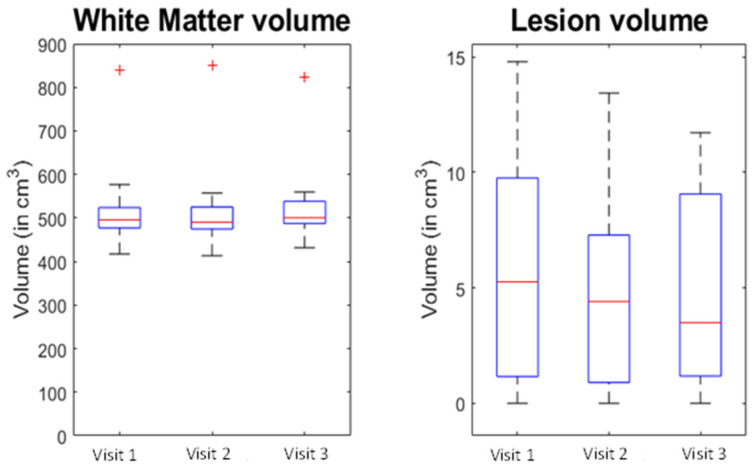
Change in white matter and lesions volumes (7T MPRAGE) during the study. Visit 1: baseline. Visit 2: 6 months. Visit 3: 12 months. The red line is the median, with the bottom and top edges of the box indicating the 25th and 75th percentiles, respectively. The whiskers extend to the most extreme data-points not considered outliers, and the red cross is showing the individual outliers.

No new lesions were detected on T1 images, and no T1 gadolinium-enhancing lesions were noted at V2 and V3 for any of the participants. No significant change was detected in the volume of the WM tissue type (F = 1.378, *p* = 0.280) or for the lesion tissue type (F = 2.822, *p* = 0.089) between the different visits (Figure 2).

**Figure 3:**
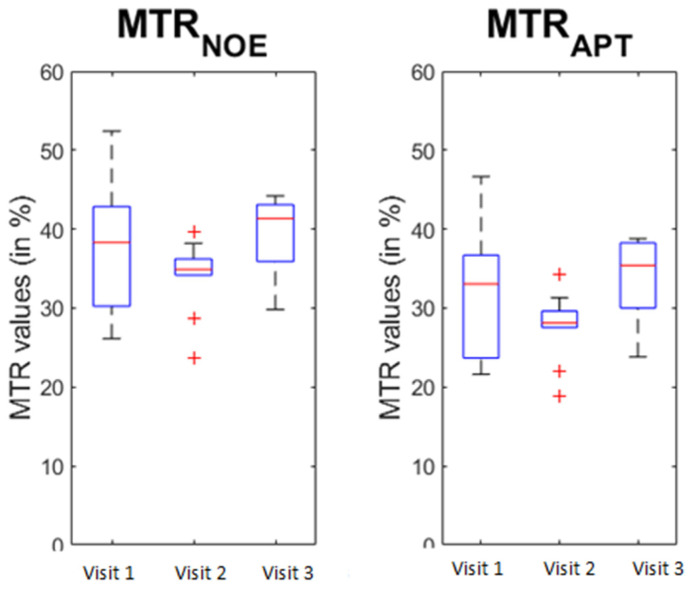
Change in the MTR values during the study. Visit 1: baseline. Visit 2: 6 months. Visit 3: 12 months. MTR: magnetisation transfer imaging. MTRAPT: amide proton transfer. MTRNOE: nuclear Overhauser effect. The red line is the median, with the bottom and top edges of the box indicating the 25th and 75th percentiles, respectively. The whiskers extend to the most extreme data-points not considered outliers, and the red cross is showing the individual outliers.

The Mauchly’s test of sphericity indicated that the assumption of sphericity was not violated for WM, GM, lesion, and brain volume. Similarly, no significant T1 change was visible in the NAWM between the different visits (F = 0.067, *p* = 0.935). Changes were detected for MTRNOE (F = 4.7, *p* = 0.025) and MTRAPT (F = 6.181, *p* = 0.01) across the repeats. When looking at a pairwise comparison between the different visits, significant differences were detected between visit 2 and visit 3 for both MTRNOE (*p* = 0.01) and MTRAPT (*p* = 0.004), showing an increase in MTR in both cases (Figure 3).

Note that T1, MTRNOE, and MTRAPT passed the Mauchly’s test for sphericity as well (*p* = 0.162, *p* = 0.392, and *p* = 0.373, respectively).

**Figure 4:**
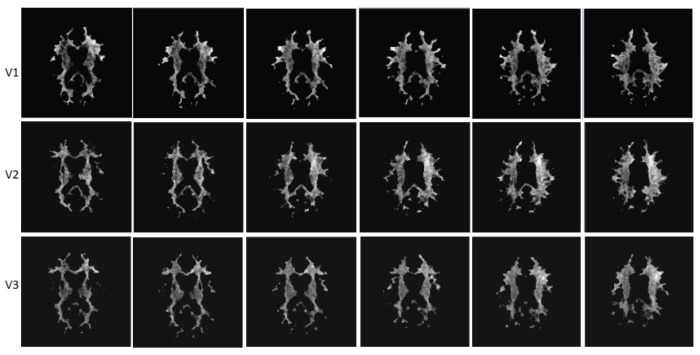
Sample CEST images of NWAM from a study participant (different anatomical levels). Visit 1: baseline. Visit 2: 6 months. Visit 3: 12 months.

A sample of CEST masks of NAWM (images from different anatomical levels for each visit) from one study participant is presented in Figure 4.

## 4. Discussion

In this prospective, open-label, observational study, we show that natalizumab treatment over 12 months is associated with disease stability and with no detected microstructural degradation in the NAWM assessed at ultra-high-field MRI. The difference in the MTR measures at month 6 and month 12 of natalizumab treatment was significant, suggesting a possible repair of tissue damage not visible with T1 quantification. This paralleled the expected lack of clinical and radiological worsening of conventional MRI measures of disease activity (new lesions or gadolinium-enhancing lesions). The EDSS scores improved in three patients.

Disease activity on MRI in MS is typically demonstrated by the appearance of new lesions, most frequently identified by hyperintensity of T2—weighted or FLAIR images. We have previously demonstrated [15] that lesions defined by focal hypointensities on T1-weighted or MT-weighted imaging at 7 T, as in the current study, were segmented with a sensitivity of 98% compared to segmentation on the hyperintense T2/FLAIR lesions on 3 T MRI scans obtained in the same subjects on the same day used as a gold standard.

This study confirms at 7 T the previous MRI reports at lower magnetic fields, showing that natalizumab can prevent and perhaps even reverse NAWM damage in MS. Using 3 T MRI serial DTI of NAWM during 1 year of NTZ treatment, Fox et al. observed changes in DTI measures suggestive of possible remyelination within acute MS lesions and chronic axonal degeneration regions in NAWM [24]. Natalizumab prevents the further accumulation of microstructural tissue abnormalities in both focal white matter lesions and NAWM measured at 1.5 T, compared to interferon-beta [17]. The treatment with natalizumab can reduce the extent and severity of NAWM destruction measured at 1.5 T after 1 year [25] and is able to stabilise the NAWM damage assessed at 3 T at 4 years [26]. In a phase 2 study on progressive MS patients, grey matter and NAWM MTR assessed at 3 T increased after 60 weeks of treatment with natalizumab [1].

The substantially increased signal-to-noise ratio at 7 T provides an increased sensitivity for changes in measured T1 relaxation time. The T1 relaxation time is mostly affected by structural changes or inflammatory oedema [27]. Therefore, the lack of change in T1 relaxation times during 1 year of treatment with natalizumab in our study argues against the development of either structural damage or oedema.

This is the first study investigating MTR at 7 T longitudinally with natalizumab in MS, giving more sensitivity to the detection of a possible change in tissue composition, especially when combined with the more standard quantitative T1. Whilst conventional MRI relies on the excitation of hydrogen nuclei in water molecules, magnetisation transfer imaging indirectly detects mobile species with exchangeable protons and can achieve unprecedented sensitivity at the molecular level [28]. A frequently reported magnetisation transfer effect is sensitive to the amide protons in proteins and peptides resonating downfield from water (amide proton transfer, reflected by MTRAPT) [29]. It has been suggested that different stages of tissue injury in MS translate into different MTRAPT values, with an increased APT signal due to protein degradation owed to axonal damage in the NWAM prior to the development of visible lesions, and a decrease in APT signal with axonal transection in advanced MS [11]. In both cases, MTRAPT reflects the change in APT signal overlaid onto the MTR signal. Similarly, sensitivity to NOE (MTRNOE) is increased at high field and can reflect a change in myelination even with high-resolution images [30]. The NOE-mediated MTRNOE signal is attributed to aliphatic and olefinic protons in mobile proteins and provides distinct information about tissue composition. A significant change was detected between the three measurements during the first 12 months of monthly treatment with natalizumab in our study, although a significant difference could only be detected between visit 2 and visit 3 in both MTRAPT and MTRNOE signal, suggesting an increase primarily in MTR. Altogether, these findings suggest an improvement in the white matter microstructure, possibly in part representing remyelination, after 1 year’s treatment with natalizumab. In a recent study (Preziosa et al., 2019 [18]), natalizumab stabilised but did not improve MTR after 24 months’ treatment, while fingolimod increased the MTR at 24 months, suggesting a remyelinating effect. However, as the current study was performed on an ultra-high-field scanner, it is possible that subtler improvements missed at 3 T in the above-mentioned study were detected in our study. Injury-sensitive magnetisation transfer MRI may complement conventional MRI to monitor the response to MS treatments.

The main limitations of our study reside in a lack of a control group, and the small number of subjects. However, the fact that we obtained results with the small number of participants that are congruent with those of larger studies performed at lower field strengths [1,25] attests to the higher sensitivity of ultra-high-field MRI to microstructural changes, which can reduce the numbers needed in clinical investigations. Another potential limitation, the absence of T2/FLAIR images, was circumvented by identifying lesions using MPRAGE [15]. Finally, a more quantitative assessment of APT and NOE signal using more complex methods and imaging [31] would help in separating the NOE and APT changes from the standard MTR effects.

An extended duration of follow-up with serial ultra-high-field MRI monitoring would be necessary to confirm the longer-term sustained stability of NWAM in people with active MS under natalizumab.

## 5. Conclusions

In this study, we show that the radiological stabilisation of brain damage in people with active MS with natalizumab is real as, even at ultra-high field, a progression of the structural damage from MS is not detected. Our findings of stable T1 relaxation times, and the demonstration, for the first time at 7 T MRI, of improved MTRAPT and MTRNOE in normal-appearing brain regions of patients with active MS argue against a diffuse or occult brain microstructural destruction occurring in the first year of treatment with natalizumab. Future work needs to determine whether, how, and when the NAWM changes occur in time on long-term treatment with natalizumab in patients with MS in whom MS inflammation is controlled who develop progression of MS independent of relapse activity.

## Figures and Tables

**Table 1:** Patient characteristics.

Patient Demographics (*n* = 12)	
Age (years, standard deviation)	34.2 (7.2)
Sex (no. of subjects, %)	
Female	7 (58%)
Male	5 (42%)
Ethnicity	
White British	10
Asian British	1
African British	1
Clinical characteristics	
Type of MS	
(no. of subjects, %)	
RR	12 (100%)
SP	0 (0%)
Time from last clinical relapse	>90 days
Annualised relapse rate (95% CI)	1.99 (1.93–2.02)
Mean EDSS score (range)	3.5 (1–7)
Lesion volume mm^3^	
(average, standard deviation)	6639.7 ± 4632.6

## Data Availability

The data presented in this study are available upon reasonable request from the corresponding author.
